# Feeding practice, energy, and nutrient intake adequacy among children aged 6–23 months in Southern Ethiopia: a community based cross‐sectional study

**DOI:** 10.1002/fsn3.1962

**Published:** 2020-10-24

**Authors:** Beshadu Bedada Feyisa, Gosaye Mekonen Tefera, Bilal Shukur Endris, Tamene Taye Asayehu, Seifu Hagos Gebreyesus

**Affiliations:** ^1^ Department of Public Health College of Medicine and Health Science Ambo University Ambo Ethiopia; ^2^ Department of Pharmacy College of Medicine and Health Science Ambo University Ambo Ethiopia; ^3^ School of Public Health College of Health Science Addis Ababa University Addis Ababa Ethiopia; ^4^ Department of Food Science and Applied Nutrition College of Applied Sciences Addis Ababa Science and Technology University Addis Ababa Ethiopia

**Keywords:** children aged 6–23 months, complementary food, energy and nutrient intake, Ethiopia, feeding practice

## Abstract

After 6 months, children require increased food and nutrient intake from complementary food for their growth and development. However, quantitative data on macro and micronutrient intakes from complementary food is limited. Thus, this study is designed to identify the adequacy of energy and micronutrient intake from complementary foods among children aged 6–23 months and to characterize current feeding practice in Southern Ethiopia. A community‐based cross‐sectional study was conducted from February to March 2016. Simple random sampling was used to recruit 190 mothers/primary caregivers of children aged 6–23 months. A repeated interactive multiple‐pass 24‐hr recall survey was used to assess' food and nutrient intake of children. Complementary food was low in animal sources, fruits, and vegetables. Most of the children (94.7%) consume grain, roots, and tubers. Vitamin A‐rich fruits and vegetables are consumed by 71 (37.8%) children. Very few (1.6%) children consume iron‐fortified food. Median protein intake exceeds the estimated requirement from complementary food. Except for vitamin B2 and B6, intake of energy and micronutrient were below world health organization (WHO) recommendations among children aged 9–23 months. In conclusion, infant and young child feeding practices in Butajira district did not conform to recommendations. Intake of energy and micronutrient from complementary food among children aged 6–23 months in Butajira district was inadequate. Consumption of a diverse diet by including animal source food (ASF) such as poultry, organ meat, chicken liver, beef, fruits, and vegetables is needed to fill the nutrient intake gap among the study participant.

## Graphical abstract

This article assess the feeding practice and energy and nutrient intake of children aged 6–23 months the study found that feeding practice does not conform to the international recommendation. Energy and nutrient intake of these children is also inadequate.

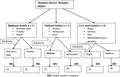

## 1 INTRODUCTION

Children between 6 and 23 months are the most vulnerable to malnutrition, because of the transition from total exclusive breastfeeding to solid foods. This period is of particular importance because this is the period when infants and young children experience rapid growth and development. During this period, growth faltering and micronutrient deficiencies are highly prevalent owing to children's high nutrient demand relative to their energy and micronutrient intakes (WHO, 2019). This transition stage presents with its challenges of complementary feeding that are linked to dietary diversity, dietary quality, food safety, and energy density (Dewey, 2003).

To combat the challenge of complementary feeding, the world health organization (WHO) recommends feeding infants and young children with a variety of complementary foods including meat, poultry, fish or eggs, as well as vitamin A‐rich fruits and vegetables every day (WHO, 2010). It also developed guiding principles for complementary feeding practices and behaviors because of their overwhelming impact on subsequent growth, health, and cognitive development during early childhood (Dewey, 2003).

Globally, the complementary feeding practice is far from the WHO recommendation, and only 28.9% of children aged 6–23 months fulfill WHO criteria for a minimum acceptable diet (Micha et al., 2020). In developing countries, complementary food fed to children 6 – 23 months had low quality and low micronutrient density (Dewey et al., 2006). Diet of the population including the diet of infant and young children in sub‐Saharan African countries is frequently deficient in energy and micronutrient leading to micronutrient deficiency disorders like anemia, iodine deficiency disorder (IDD), zinc deficiency, and vitamin A deficiency (Müller & Krawinkel, 2005).

In Ethiopia, the diet of infants and young children was predominantly cereals and legume‐based with limited consumption of nutrient‐dense animal source food, fruits, and vegetables (Central Statistical Agency, 2016). Because of the different bioactive anti‐nutrient factors such as phytates, oxalates, and the forms of the nutrients in plant‐based diets, the bioavailability of nutrients like zinc (Zn) and iron (Fe) is low (WHO/FAO, 2004). Thus, energy and micronutrient such as vitamin A, C, and zinc density in complementary food were inadequate for children aged 6–23 months in Ethiopia (Baye et al., 2015; Gibson et al., 2009). Complementary food feeding practice also not conforms to WHO recommendations. Only 7% of children aged 6–23 months in Ethiopia meet the minimum acceptable dietary standards (Central Statistical Agency, 2016).

Currently, there is speculation that being in health and demographic surveillance system (HDSS) sites have better health indicators compared to populations not under surveillance because the repeated data collection and measurement may function as a passive intervention resulting in behavioral change. Populations in HDSS areas are often exposed to studies that may provide interventions (Ye et al., 2012).

Despite there is speculation that being in HDSS has a positive effect on health indicators (Afework et al., 2014; Ye et al., 2012), the perinatal maternal mental disorder in Ethiopia (P‐MaMiE) birth cohort conducted in Butajira HDSS reported a high prevalence of under‐nutrition among infants in the study area (Medhin et al., 2010). Poor caring and feeding practice of the breastfed child (Dewey, 2003) as well as prenatal maternal nutritional status and household sanitary facility (Medhin et al., 2010) are the major factors that predispose children to energy and micronutrient deficiency in Butajira district.

There are limited data on whether child feeding practice may adhere to the WHO guiding principle for complementary feeding in the Butjira HDSS site. Moreover, there were limited data on quantitative energy and micronutrient intake adequacy among children aged 6–23 months. Such data are important to design appropriate interventions to enhance the quality of complementary food and feeding practice during the vulnerable period of transition from breastfeeding to the family diet. It helps to understand the specific nutrient gap in young children's diets that is essential to understand how to improve their diets, which can affect child growth and development. Hence, this study aimed to assess, (a) complementary food feeding practice according to WHO guiding principle for complementary feeding, (b) Energy and selected micronutrient intake adequacy, and prevalence of inadequate intake among children aged 6–23 months in Butajira HDSS of southern Ethiopia.

## 2 METHODOLOGY

### 2.1 Study design and study area

A community‐based cross‐sectional study design was conducted from February to March 2016 in a subsistent farming community in the Butajira district among children aged 6–23 months. The site is also known as Butajira Rural Health Program (BRHP) or Butajira HDSS, which was initiated in 1986. This district is found 130 km away to the south of Addis Ababa. It consists of 10 surveillance villages which were sampled in 1986 based on probability proportional to size technique from 86 kebeles of, Gurage Zone, in the south nation, nationalities and people (SNNP) Regional State in southern Ethiopia. Kebele is the smallest administrative unit that consists of about 5,000 population. The district has three agro‐ecology (lowland, midland, and highland). Maize, sorghum, false banana, and stew made from kale are the staple food in the area. The main means of livelihood in the district is rain‐dependent agriculture. It is characterized by the production of subsistence crops (mainly cereals, legumes, vegetables, fruits) and some cash crops such as Khat (Catha edulis).

### 2.2 Sample size and sampling technique

The sample for the study was determined by using a single population proportion formula based on the following assumption.

*p* = 4% the proportion of 6–23‐month‐old children who fed appropriately according to WHO Infant and young child feeding (IYCF) guideline (Central Statistical Agency, 2012).

*p* = 8% prevalence of inadequate dietary zinc intake (Engle‐Stone et al., 2014).

*Z* = is the standard normal score set at 1.961 (95% confidence interval).

*d* = is the margin of error to be tolerated (5%).

Design effect = 1.5.

Nonresponse rate = 10%.

A large sample was selected after it was calculated for both feeding practice and adequacy (Table 1).

**TABLE 1:** Sample size calculation for the study conducted among children aged 6–23 in Butajira HDSS, SNNP region, 2016

Appropriate complementary feeding practice in Ethiopia *p* = 4%	$n=\frac{{\left(Z\alpha /2\right)}^{2}p\left(1‐p\right)}{d^{2}}=\frac{\left(1.96\right)2\times \left(0.04\times 0.96\right)}{{\left(0.05\right)}^{2}}=\;59$ considering design effect 1.5:1.5 * 59 = 89
prevalence of inadequate zinc intake *p* = 8%	$n=\frac{{\left(Z\alpha /2\right)}^{2}p\left(1‐p\right)}{d^{2}}=\frac{\left(1.96\right)2\times \left(0.08\times 0.92\right)}{{\left(0.05\right)}^{2}}=\;113$ considering design effect = 1.5:1.5 * 113 = 170

The large sample (*n* = 170) was taken and considering 10% of nonresponse rates a total of 190 mothers or caregivers of 6‐ to 23‐month‐old children participated in the study. Forty mothers were interviewed for second‐day 24‐hr dietary recall resulting in 230 interviews. Two interviews were dropped because of incomplete dietary descriptions.

**FIGURE 1:**
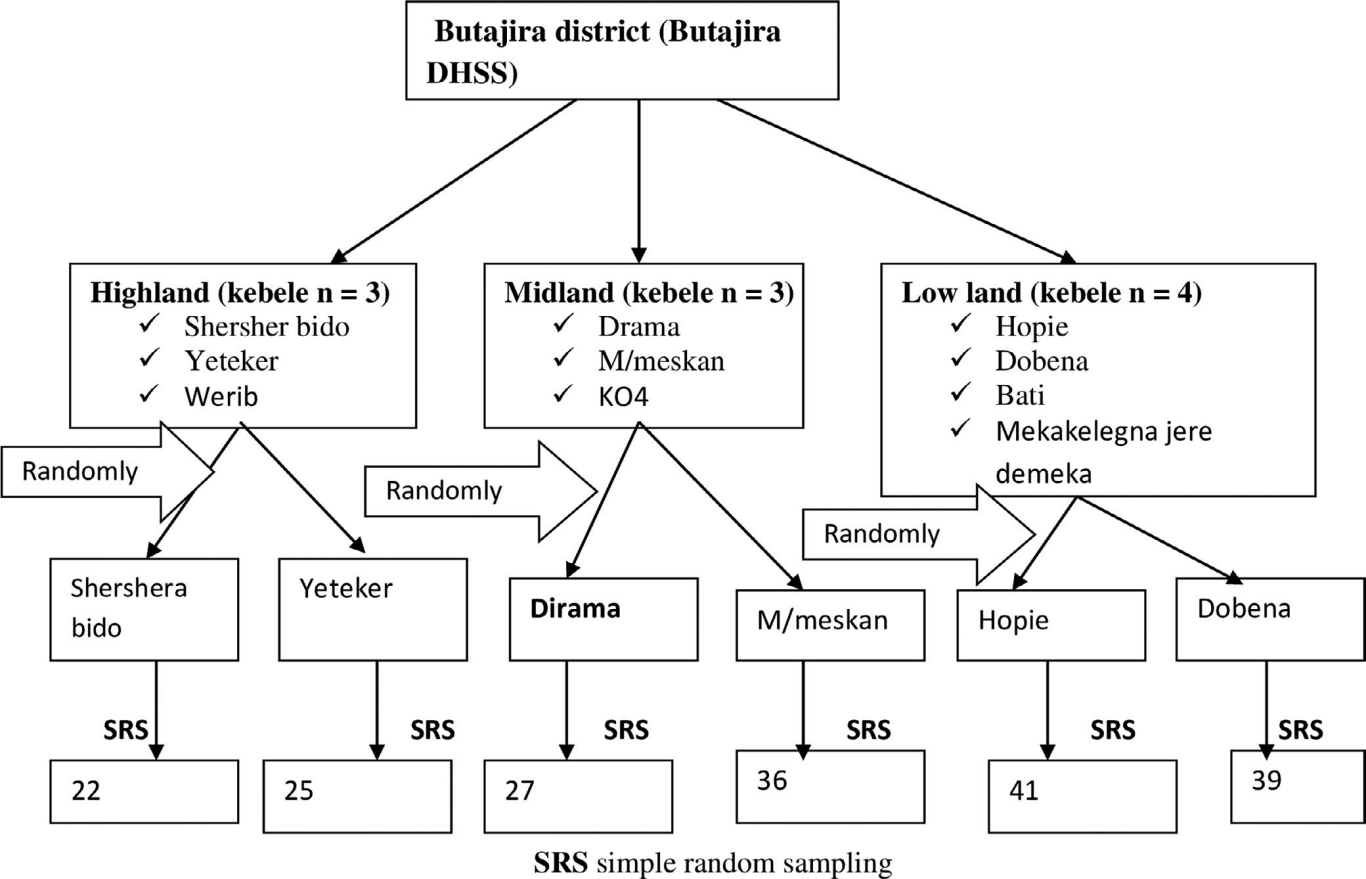
Sampling procedure of children in Butajira district, 2016

The district was divided into three agro‐ecologic zones, and two villages from each agro‐ecology were randomly selected for this study (Figure 1). The study participant was identified by a simple random sampling technique from each selected village. Lists of households in the district with children 6–23 months (birth from February 2014 to August 2015) were obtained from the BRHP database. This list was used as a sampling frame to select the study participant.

Ethical clearance was obtained from, School of Public Health, College of Health Sciences, Addis Ababa University, Research, and Ethics Committee. Informed verbal consent was obtained from each study participants, before the interview and after an explanation of all the study's purpose and procedure. This study was done in accordance with the ethical guidelines of the Declaration of Helsinki.

### 2.3 Operational and standard definition

Minimum dietary diversity score: The proportion of children 6–23 months of age who receive four or more food groups during the previous day (WHO, 2010).

Minimum meal frequency: The proportion of 6–8 months and 9–23 months children who receive meals at least two times and three times per day, respectively (WHO, 2010).

Minimum acceptable diet: The proportion of breastfed children 6–23 months of age who had at least the minimum dietary diversity score and the minimum meal frequency per day (WHO, 2010).

Adequate intake: Intake of nutrient assessed in this study (energy, protein, vitamin B1, B2, B6, folate, vitamin C, iron, calcium, and zinc) were adequate if median intake of these nutrients meets estimated requirement based on recommended nutrient intake (RNI) for infant 6–11 months and estimated average requirement (EAR) for toddler‐aged 12–23 months (Gibson et al., 2009; Gibson & Elaine, 1999).

Prevalence of inadequacy: The proportion of children who receive nutrients less than the estimated average requirement after adjustments for intra‐individual intake variation (Asayehu et al., 2017; Gibson & Elaine, 1999).

Portion size of egg estimated in a piece: The average weight of ten different sized egg's edible portions was used to estimate the portion consumed.

Portion size of an avocado estimated in the piece: An edible portion of 10 medium‐sized avocados was weighed, and the average weight was taken for analysis.

### 2.4 Exclusion criteria

Children who have an illness at the time of data collection reported by mother/caregiver, children whose mother or caregivers are on unusual situations such as death or wedding, and those who did not initiate complementary food were excluded from this study. Since only four none breastfeed children were identified, they were excluded and other samples of breastfed children were selected.

### 2.5 Assessments of dietary intake

An interactive, multiple‐pass 24 hr dietary recall questionnaire adapted and validated for use in developing countries (Gibson & Elaine, 1999) was used to collect data on dietary intake.

Three days before the interview, infant and young child recipe and photo of equipment commonly used to serve food for children aged 6–23 months were collected to purchase and arrange similar equipment from the local market for data collection. The recipe was used to probe for food intake and describe ingredients of listed food during data collection. The equipments were labeled and calibrated using the electronic Seca scale by cooking similar food using a similar cooking method. On the interview day, the participants were requested to report all foods that they served for their child on the previous day, using a multiple‐pass method. The total sample size was allocated proportionally to the seven days of the week, and the dietary data were collected throughout all seven days of the week to account for the day‐to‐day variation of food consumption. Other information such as socio‐demographic and feeding practice was collected by the same data collector using a standardized questionnaire.

The second recall was repeated on 20% of randomly selected study participants on the nonconsecutive day by other interviewer, and it was used to adjust for the day‐to‐day variation of nutrient intakes of the study participants. All the recall days were arranged on nonspecial occasions such as holiday, feast day, death occasion in the household, or fasting time.

To estimate portion size, the interviewers were equipped with calibrated locally available labeled infant feeding equipment, a salted replica of common local infant and young child food, and a food weighing scale. We used about four dishes of the salted replica of common infant and young child food. These include thin gruel made from the flour of mixture of different cereals and legumes, Injera/Ethiopian flatbread, a flatbread made from whole grain maize, and stew made from Ethiopian kale. During dietary recall, each participant was asked to put the amount of food that is equivalent to the actually eaten, if actual food is available or from a salted replica on food weighing scale. If actual food is not available in the house, we asked them to borrow from neighbors. Otherwise, we asked to estimate the portion of food that the child actually eats using equipment handled by the data collector. For purchased food, the monetary values and labeling/brand of the food were asked. The participants were also asked to estimate the portion of leftover if any, using the same method as they estimate the intake. If there is no leftover, the usual amount given to the child was calculated for total energy and nutrient intake. Every participant was probed for a snack, fluid, and outdoor consumption of food after they complete recall.

To estimate the portion size of boiled potato, raw potato was weighed during data collection and the portion consumed was estimated by multiplying with a yield factor of 112% (McCarthy, 2002). All collected dietary data were converted to grams of food consumed.

Ethiopian food composition table (FCT) (Ethiopian Health & Nutrition Research Institute, 1998) was used to calculate the nutrient values of food. Uganda (Hotz et al., 2012) and USDA Sr 26 food composition table (Haytowitz & Holden, 2012) were used to calculate the nutrient value of food that is not found on the Ethiopian FCT. Since there was no nutrient value of boiled milk, raw milk was taken and converted by nutrient retention factor if milk was boiled approximately 10 min (Ahuja, 2007). The nutrient composition of thin gruel made from the flour of mixture of different cereals and legumes is calculated from the flour in Ethiopian FCT (Ethiopian Health & Nutrition Research Institute, 1998) considering the nutrient retention factor. The dietary data were analyzed and converted to the amount of nutrient and energy intake per day by using software called food processor (version 8.1). To calculate total retinol equivalent (RE) (mcg) in the diet, beta carotene (mcg) was divided by 12 (retinol activity equivalent (RAE) factors of 12:1 for β‐carotene) and added to RE mcg (WHO/FAO, 2004). Low bioavailability was assumed to asses' adequacy of iron and zinc. For those who have a two‐day dietary recall, the average was taken to assess intake from complementary food.

### 2.6 Assessment of dietary adequacy

Nutrient requirements from complementary food were estimated by subtracting the nutrient concentration of breast milk from RNI developed by WHO/FAO joint expert consultation (WHO/FAO, 2004) and from EAR developed by the institute of medicine (Institute of Medicine, 2001) and report of FAO/WHO expert consultation (FAO/WHO, 2001) for children aged 6–11 months and 12–23 months, respectively, assuming average breast milk intake and nutrient composition (WHO, 1998). The estimated protein requirement from complementary food was taken from WHO (1998), and energy requirement from complementary food was based on US longitudinal data (Butte et al., 2000). After obtaining the estimated requirement from complementary food, we compare the median intake of children included in our study with an estimated requirement from the table of nutrient reference level (Gibson & Elaine, 1999).

### 2.7 Assessment of usual nutrient intake and prevalence of inadequate intake

To assess usual nutrient intake the first day, 24‐hr dietary recall was adjusted by using the second‐day recall to account for, with‐in‐person day‐to‐day variation of food intake using the Intake Monitoring Assessment and Planning Program (IMAPP) software (Iowa State University, 1995–2015). The software adjusts for intra‐individual day‐to‐day variation of usual nutrient intake to estimate the prevalence of inadequacy. Nutrient intake from complementary food and breast milk was imported to the IMAPP software to estimate the usual nutrient intake of the study participants. Prevalence of inadequate micronutrient intake, namely vitamin A, B1, B2, B6, folate, calcium, and zinc was analyzed for children aged 12–23 months based on the EAR cutoff method by comparing usual nutrient intake with the EAR cutoff point.

### 2.8 Assessment of feeding practice

Child feeding practice was assessed whether mothers and/or caregivers were adhering to the WHO guiding principles for complementary feeding of the breastfed child (Dewey, 2003).

### 2.9 Data quality management

Experienced four female data collectors and two supervisors who were fluent in the local language were recruited and trained for three days in‐classroom setting. This was followed by pretesting of the questionnaire on 16 mothers of children 6–23 months who were comparable to actual study participants. These mothers do not participate in the actual study. The principal investigator and field supervisor rechecked for completeness and consistency of the questionnaire immediately after the interview at field level and during submission.

### 2.10 Data analysis

The socio‐demographic characteristics of the mother and child as well as the feeding practice of the child were entered into epidata version 3.1. Then, the data were exported to STATA version 12 for analysis. Continuous variables were presented using mean ± *SD* or median (IQR) after testing for normality using skewness and kurtosis test. The categorical variables were presented using frequency and percentages.

## 3 RESULTS

### 3.1 Socio‐demographic characteristics of the respondent and the child

Most of the respondents (98.40%) were biological mothers of the children. The mean age of the mothers was 30.50 (*SD* ± 6.31) years. Almost all (99.50%) mothers were married, and 149 (79.30%) were housewives. One hundred and eight (57.40%) mothers had no formal education. Most (71.30%) respondents were Muslim followed by Orthodox Christianity which accounted for 77 (16.30%) of the total sample. Around two‐thirds of the study, the population was from the Gurage ethnic group followed by Silte which accounted for 45 (23.40%) of the total sample. More than half of the mothers cannot read and write. The mean age of the child was 13.50 **(** *SD* ± 5) months. About one hundred eight (57.50%) studied children were male, and 80 (42.50%) were female (Table 2).

**TABLE 2:** Socio‐demographic characteristics of the study participants in Butajira HDSS, SNNP region, 2016 (*n* = 188)

Characteristics	Frequency (*n* = 188)	Percent
Child age group (months)		
6–8	42	22.3
9–11	35	18.6
12–23	111	59.0
Child sex		
Female	80	42.5
Male	108	57.5
Relation of the child with respondent		
Mother	186	98.4
Residence		
Rural	178	94.7
Urban	10	5.30
Respondent's age (year)		
15–19	27	14.4
20–24	56	29.8
25–29	46	24.6
30–34	36	19.1
35–39	18	9.6
40 and above	5	2.7
Marital status		
Married and living together	187	99.5
Divorced	1	0.50
Religion		
Muslim	134	71.3
Orthodox Christian	31	16.3
Protestant	23	12.2
Ethnicity		
Gurage	117	62.2
Silte	45	23.4
Kontoa	22	11.7
Others	4	2.10
Level of education		
Cannot read and write	108	57.4
Primary school	74	39.4
Secondary school and above	5	2.6
Occupation		
Housewife	149	79.3
Merchant	21	11.2
Small business ‐local drink seller	11	5.9
Framer	4	2.1
Other	3	1.6

### 3.2 Complementary food feeding practice

**FIGURE 2:**
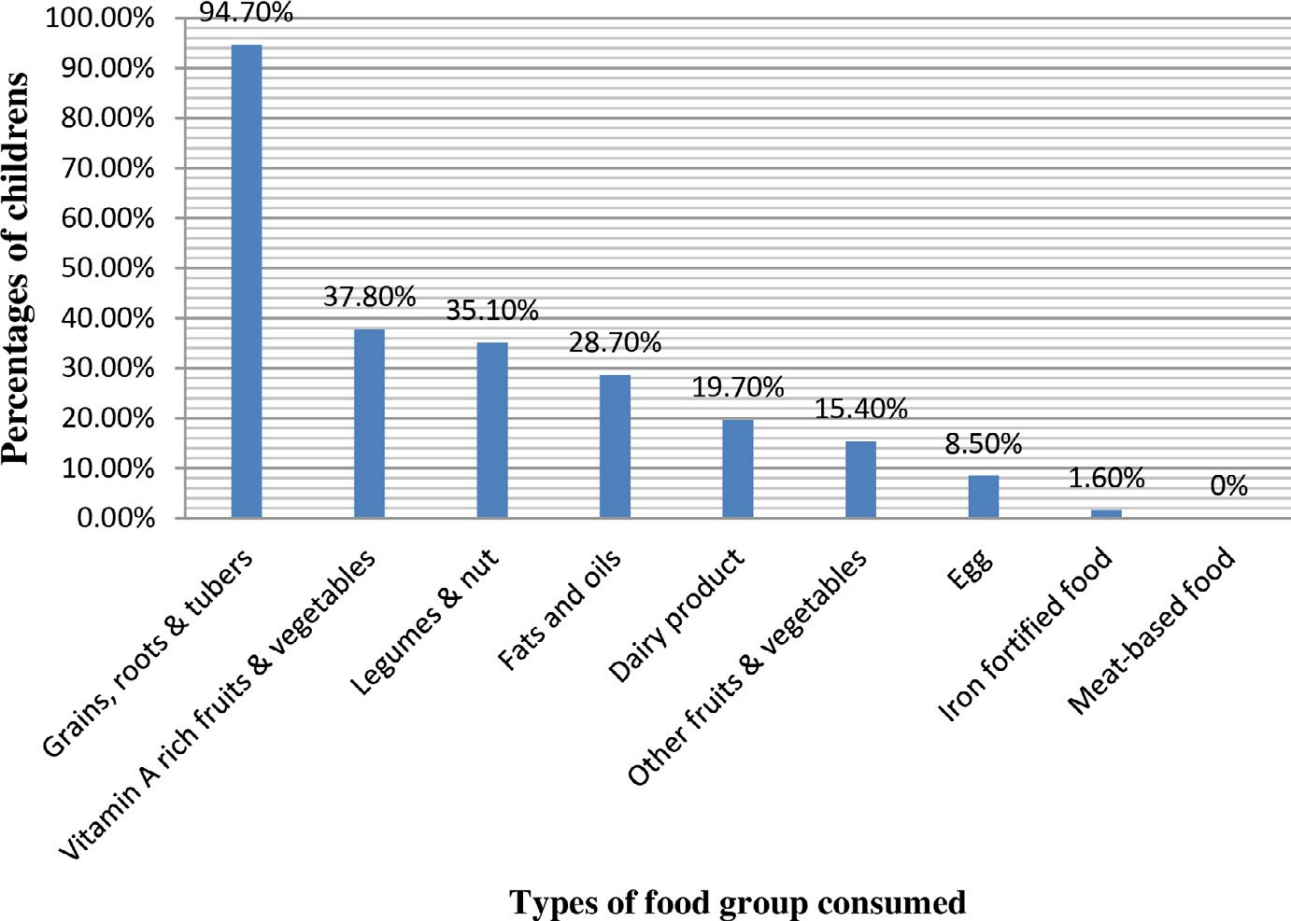
Food group consumed by children aged 6–23 months in Butajira HDSS, Ethiopia, 2016

Grains, roots, and tubers were the dominant food groups consumed by most (94.70%) of the children. Vitamin A‐rich fruits and vegetables were the second most widely consumed food groups by 37.80% of the study participant. Consumption of eggs and dairy products was relatively low (8.50%) and none (0%) of our study participants consume meat‐based food. Consumption of commercial iron‐fortified baby food was low 3 (1.60%) (Figure 2).

The mean meal frequency of these children was 3 ± 1.02 over 24 hr before the interview. About 79.80% of these children were fed complementary food minimum number of times with respect to their age according to WHO recommendation for IYCF practice. However, only 2.70% of children were fed according to minimum standards with respect to food diversity (four or more food groups) (Table 3).

**TABLE 3:** Complementary feeding practices among children 6–23 months in Butajira HDSS, Ethiopia, 2016

Characteristics	Frequency	Proportion (%)
Minimum dietary diversity		
Consumed ≥4 food groups	5	2.7
Consumed <4 food groups	183	97.3
The scale of the dietary diversity score		
Low (0–2 food group)	136	72.3
Medium (3–4 food group)	150	26.6
High (>4 food group)	2	1.1
Minimum meal frequency (received food the minimum number of times)		
Yes	150	79.8
No	38	20.2
Meal frequency (mean ± *SD* *)	3 ± 1.02	
Received a minimum acceptable diet		
Yes	5	2.7
No	183	97.3
Feeding practice by age group		
Minimum dietary diversity score (≥4 food group)		
6–8 months	0	0.00
9–11 months	2	5.70
12–23 months	3	2.70
Minimum meal frequency		
6–8 months	33	78.60
9–11 months	19	54.30
12–23 months	98	88.30
Minimum acceptable diet		
6–8 months	0	0.00
9–11 months	2	5.70
12–23 months	3	2.70

* *SD*, standard deviation.

### 3.3 Adequacy of energy and nutrient intake from complementary food for breastfed children

The median energy intake from complementary food for all of the three age groups was below the recommended energy requirement. The median protein intake of children who participated in this study exceeds the estimated protein requirement from complementary food. The median intake of most of the micro‐nutrients assessed in this study was less than the corresponding estimated requirement from complementary food. However, folate intake of 9–11 months and vitamin B2 and B6 intake of 12–23 months children exceeds the recommended intake (Table 4).

**TABLE 4:** Nutrient intake from complementary food for breastfed children in Butajira HDSS, Ethiopia, 2016

Nutrient	Age group of children								
	6–8 months			9–11 months			12–23 months		
	Median intake (IQR)	RNI	% <RNI (95% CI)	Median intake (IQR)	RNI	% <RNI (95% CI)	Median intake (IQR)	EAR	% <EAR (95% CI)
kcal/d	125 (115.2, 157.5)	202.0	85.70 (0.75, 0.97)	145.3 (145.2, 162.4)	307	97.10 (0.90, 1)	155.5 (155.3, 155.8)	548	100
Protein (g/d)	2.67 (1.5, 5.9)	2.0	28.60 (0.14, 0.43)	5.70 (3.30, 8.70)	3.10	22.8 (0.1, 0.4)	8.78 (6.1, 12.6)	5	31.70 (0.30, 0.57)
Vit A‐RE (mcg/d)	6.10 (0.5, 17.4)	69.7	92.90 (0.85, 1.00)	25.20 (0.15, 102.2)	91.70	74.30 (0.6, 0.9)	89.9 (24.1, 912.8)	125.2	53.20 (0.40, 0.63)
Vitamin B1 (mg/d)	0.05 (0.0, 0.2)	0.16	78.60 (0.66, 0.92)	0.10 (0.0, 0.2)	0.17	80 (0.7, 0.9)	0.10 (0.1, 0.2)	0.28	83 (0.76, 0.9)
Vitamin B2 (mg/d)	0.06 (0.02, 0.14)	0.17	81 ( 0.69, 0.93)	0.20 (0.10, 0.30)	0.20	54.30 (0.4, 0.7)	0.4 (0.2, 0.6)	0.21	34.23 (0.2, 0.4)
Vitamin B6 (mg/d)	0.15 (0.02, 0.74)	0.22	59.50 (0.44, 0.75)	0.20 (0.10, 0.50)	0.20	54.30 (0.4, 0.7)	0.53 (0.3, 0.8)	0.33	29.3 (0.2, 0.40)
Vitamin C (mg/d)	0.32 (0.03, 0.88)	3.60	92.90 (0.44, 0.75)	0.80 (0.00, 2.90)	5.36	85.70 (0.7, 1.0)	1.6 (0.6, 3.8)	8.04	100
Folate (mcg/d)	9.6 (2.14, 31.06)	23.0	66.67 (0.52, 0.84)	29.90 (7.10, 44.1)	27.60	48.60 (0.3, 0.7)	52.2 (33.9, 76.0)	73.3	73 (0.70, 0.80)
Calcium (mg/d)	16.55 (3.43, 51.9)	85.20	92.90 (0.85, 1)	54.40 (13.7, 144.2)	97.50	65.70 (0.5, 0.8)	119.9 (49.7, 239.5)	246.3	76.60 (0.70, 0.9)
Iron (mg/d)	0.20 (0.1, 0.7)	18.40	100	0.50 ( 0.20, 1.30)	18.42	100	1.2 (0.7, 10.9)	11.44	96.6 (0.95, 1)
Zinc (mg/d)	0.38 (0.15, 0.8)	7.61	99 (0.90, 1)	0.80 ( 0.20, 1.40)	8.02	100	1 (0.90, 2.40)	6.24	100 (0.90, 1.0)

Note

IQR—inter quartile range, RNI—recommended nutrient intake, and EAR—estimated average requirement, RNI is according to WHO/FAO expert consultation (WHO/FAO, 2004) EAR is according to estimated need developed by institute of medicine. For children, aged 12–23 months except for zinc which is from WHO guideline for food fortification with micronutrient (WHO, 2006). Estimated requirement for iron of children aged 12–23 months depends on WHO/FAO RNI (WHO/FAO, 2006) because there is no EAR for this age group. Estimated protein requirement from complementary food was taken from WHO, 1998 (WHO/FAO, 1998). Energy requirement from complementary food was based on US longitudinal data (Butte et al., 2000).

### 3.4 Prevalence of inadequacy

About 68.20% of toddler‐aged 12–23 months were at risk of protein inadequacy. Similarly, about 84.40%, 33.80%, 27%, and 70.70% of these children were at risk of inadequacy for vitamin B1, B2, B6, and folate, respectively. The prevalence of inadequacy of calcium and zinc was 76.80% and 67.30%, respectively. All the sampled children aged 11–23 months have adequate intake for vitamin A (Table 5).

**TABLE 5:** Prevalence of inadequacy of selected nutrient intake of children aged 12–23 months in Butajira HDSS, Ethiopia, 2016

Nutrient	EAR	Intake at 5th	Median	Intake at 95th	Prevalence of inadequacy
Protein (g)	10.9	2.80	8.80	237	68.2%
Vit A‐RE (mcg)	210	411.9	836.1	32,886	0%
Vitamin B1 (mg)	0.4	0.14	0.22	0.84	84.4%
Vitamin B2 (mg)	0.4	0.24	0.50	1.56	33.8%
Vitamin B6 (mg)	0.4	0.16	0.58	7.56	27%
Folate (mcg)	120	59.0	96.8	200.4	70.7%
Calcium (mg)	400	164.78	259.1	827.11	76.8%
Zinc (mg)	6.9	1.02	2.19	5.41	67.3%

Note

EAR adopted from those proposed by the institute of medicine (Institute of Medicine, 2001) and report of FAO/WHO expert consultation (FAO/WHO, 2001). EAR for zinc is adopted from (WHO/FAO, 2006).

## 4 DISCUSSION

In developing countries, identifying the specific nutrient gaps and feeding practice among young children is essential to provide evidence‐based intervention to improve child nutritional status. Thus, this community‐based cross‐sectional study aimed to identify complementary food feeding practice, adequacy of energy, and nutrient intake of infant and young children aged 6–23 months old in Butajira HDSS. Most children's feeding practice does not conform to WHO/UNICEF recommendations for child feeding. Cereals, roots, and tubers were the dominant food group consumed by children aged 6–23 months in Butajira district. The median energy and most nutrient intake were low for children aged 6–23 months in the study site.

Similar to the finding of this study previous study done in a different part of Ethiopia also identified that cereals, roots, and tubers were the dominant complementary food (Baye et al., 2013; Mekbib Ergib et al., 2014).

Vitamin A‐rich fruits and vegetables were the second most (37.80%) widely consumed food groups among breastfed children in the Butajira district. This result is higher than the finding of most other studies conducted in a different part of Ethiopia (Baye et al., 2013; Gatahun Agedew Eskezyiaw, 2015; Gibson et al., 2009; Mekbib Ergib et al., 2014). This may be because of differences in the study setting, variation in the agro‐ecological zone, and difference in food habit that is explained by the fact that vitamin A‐rich green leafy vegetables (kale) and pumpkin are planted in this particular study area during this study period. This may also be contributed to the consumption of vitamin A‐rich vegetable as homegrown fruits and vegetables have a positive effect on the consumption of food rich in vitamin A (Ecker et al., 2010).

Animal source food can fill multiple micronutrient deficiencies even at a small volume of intake (Neumann et al., 2003), and it is recommended for children from developing countries more than those from developed countries (Rivera et al., 2003). This study depicted the consumption of meat‐based foods that have good bio‐available micro‐nutrients such as iron and zinc was nil. Other studies conducted in Ethiopia including the Ethiopian demographic and health survey (EDHS), (Central Statistical Agency, 2016) reported consumption of meat‐based food was very low (Baye et al., 2013; Gibson et al., 2009; Tessema et al., 2013).

A few (8.50%) of children included in this study consume egg. A similar scenario was reported by EDHS (Central Statistical Agency, 2016) and a study done in Ghana (Gyampoh et al., 2014). Perhaps this figure is higher than the finding of the study conducted in Southern Ethiopia, which reports very few children consumed egg (Tessema et al., 2013). This may be because of differences in the study setting. The current study was conducted in Butajira HDSS. Being in the HDSS and the current health extension services may also help mothers to give eggs for their children in our study area. There is evidence that being in HDSS has a positive influence on the health care of the local community (Afework et al., 2014; Ye et al., 2012).

Dairy product consumption was also very low. A similar result was reported by other studies conducted in different parts of Ethiopia (Baye et al., 2013; Mekbib Ergib et al., 2014).

Even though the majority (79.80%) of infant and young children in Butajira district received minimum meal frequency, only a few (2.70%) of 6‐ to 23‐month‐age children were received the recommended number of food groups (>4 food group). This indicates limited food groups, particularly cereals, were consumed frequently. Perhaps the finding of this study was somewhat lower than the finding of other studies conducted in Ethiopia (Gibson et al., 2009; Gyampoh et al., 2014; Khanal et al., 2013; Mokori & Orikushaba, 2012; Tessema et al., 2013). This difference result from the fact that this study validates food intake if child consumes 10 g of food group and intake less than 10 g was not analyzed for food intake. The other reason for low dietary diversity may be the presence of drought in the study area.

Meal frequency coupled with appropriate food consistency is a proxy indicator for energy intake from complementary food (WHO, 2010). This study found that even though the majority of surveyed children receive meal minimum number of times, except protein intake, energy intake from complementary food was inadequate. This may be due to the low energy density of their diet, coupled with the consumption of a small quantity of food with limited diversity. Moreover, thin gruel made of a mixture of cereals and legumes and pieces of unleavened maize bread was commonly consumed among children in the Butajira district. This inappropriate feeding practice may also contribute to inadequate energy intake. This finding was consistent with earlier studies conducted in a different region of Ethiopia (Baye et al., 2013; Gibson et al., 2009). Median protein intake from complementary food exceeds the estimated requirement from complementary food. A similar finding was reported by other studies conducted in other parts of Ethiopia (Baye et al., 2013; Gibson et al., 2009).

In contrast to the report of a study done in Sidama zone (Gibson et al., 2009), this study found that folate intake among children aged 9–11 months and riboflavin and vitamin B6 intake among children aged 12–23 months met their estimated requirements. This discrepancy may be due to variation in the study period and study area. In our study area, green leafy vegetables and stew made from dried beans were commonly consumed. On the other hand, in some parts of the study area, orange and avocado were fed for children, as these fruits were harvested and cheap during the season of our data collection.

In line with other studies conducted in northern Wallo of northern Ethiopia (Baye et al., 2013), this study found that most of the micronutrient intake from complementary food was inadequate. This might be due to low compliance with WHO guiding principles for complementary feeding. The diet of most of the study participants was grains, roots, and tubers. Their dietary diversity score is very low. There is evidence that dietary diversity score is the indicator of micronutrient adequacy (Kennedy et al., 2007; Zhao et al., 2017). Consumption of animal source food improves the micronutrient density of complementary food (Neumann et al., 2003). However, few children in the study site consume animal source food.

Iron and zinc intake from complementary food among children in the Butajira district is inadequate. A similar finding was reported by the study done in the Sidama zone (Gibson et al., 2009). This may be because none of the study children included in this study consume meat‐based food and few of them consumed iron‐fortified food. WHO/FAO joint expert consultation recommends that consumption of meat and fish increases the intake of bio‐available iron and zinc and such food has a significant amount of iron and zinc as compared to plant‐based food. On the other hand, the heme iron from meat‐based food also improves the bioavailability of iron from the rest of the diet (WHO/FAO, 2004).

In conclusion, very few children fed complementary food according to WHO guiding principle for infant and young child feeding. Although this study was conducted during the postharvesting time, energy and most nutrient intake from complementary food among infant and young children in Butajira district were below the EAR/RNI for their respective age group.

The local government and NGO's working on maternal and child nutrition should work on improvement of the quality of complementary food. The government and NGO working on rural development programs should ensure the initiation and implementation of nutrition‐sensitive agriculture at the household level to impact on dietary diversity. The promotion of small animal rearing for household consumption may also bring significant improvement in the consumption of animal source food to improve the nutrient composition of complementary food.

The existing supplementation programs and nutrition‐specific activities coverage has to be strengthened in the study area. Any possible effort should be done to increase the micronutrient intake of children in the study area. Health extension service has to be further enhanced and strengthened to promote optimal infant and young child feeding practices through the promotion of consumption of diverse diet by including ASF such as poultry, organ meat, chicken liver, beef, fruits, and vegetables. Further research is also needed to identify the effect of seasonal variation on nutrient intake and feeding practice in Ethiopia.

The findings of this study were interpreted in the context of the following strength and limitations. Experienced data collectors who had collected similar data for the assessment of nutrient intake among pregnant women in the same study area were recruited and trained for three days. We used a salted replica of local common infant and young child food to estimate portion size. To account for the effect of days of the week on the dietary intake, the final sample was proportionally allocated for all days of the week including weekend days. This study also reports the prevalence of risk of the inadequacy of nutrient intakes among children aged 6–23 months, based on usual nutrient intake distribution. Dietary data were collected twice from the subsample to account for day‐to‐day variation in nutrient intake.

Despite this strength, this study has also the following limitations. The cross‐sectional nature of this study does not allow evaluation of the seasonal variation effect on energy and nutrient intake of children. Moreover, breast milk intake is also not quantified.

## CONFLICT OF INTEREST

The authors declare that we have no conflict of interest.

## ETHICAL APPROVAL

The study's protocols and procedures were ethically reviewed and approved by Institutional Ethical Review Board of School of Public Health, College of Health Sciences, Addis Ababa University. Informed verbal consent was obtained from each study participants, before the interview and after an explanation of all the study's purpose and procedure. This study was done in accordance with the ethical guidelines of the Declaration of Helsinki.

## Data Availability

The data that support the findings of this study are available from the corresponding author upon reasonable request.
